# Two biases in incubation time estimation related to exposure

**DOI:** 10.1186/s12879-024-09433-7

**Published:** 2024-06-03

**Authors:** Vera H. Arntzen, Marta Fiocco, Ronald B. Geskus

**Affiliations:** 1 Mathematical Institute, Leiden University, Leiden, the Netherlands; 2 Biomedical Data Science, section of Medical Statistics, Leiden University Medical Center, Leiden, the Netherlands; 3 Statistics, Princess Maxima Center for Child Oncology, Utrecht, the Netherlands; 4 Centre for Tropical Medicine, Oxford University Clinical Research Unit, Ho Chi Minh City, Viet Nam; 5 Centre for Tropical Medicine and Global health, Nuffield Department of Clinical Medicine, University of Oxford, Oxford, UK

**Keywords:** Differential recall, Left truncation, Incubation time, SARS-CoV-2, Interval censoring

## Abstract

**Background:**

Estimation of the SARS-CoV-2 incubation time distribution is hampered by incomplete data about infection. We discuss two biases that may result from incorrect handling of such data.

Notified cases may recall recent exposures more precisely (differential recall). This creates bias if the analysis is restricted to observations with well-defined exposures, as longer incubation times are more likely to be excluded.

Another bias occurred in the initial estimates based on data concerning travellers from Wuhan. Only individuals who developed symptoms after their departure were included, leading to under-representation of cases with shorter incubation times (left truncation). This issue was not addressed in the analyses performed in the literature.

**Methods:**

We performed simulations and provide a literature review to investigate the amount of bias in estimated percentiles of the SARS-CoV-2 incubation time distribution.

**Results:**

Depending on the rate of differential recall, restricting the analysis to a subset of narrow exposure windows resulted in underestimation in the median and even more in the 95th percentile. Failing to account for left truncation led to an overestimation of multiple days in both the median and the 95th percentile.

**Conclusion:**

We examined two overlooked sources of bias concerning exposure information that the researcher engaged in incubation time estimation needs to be aware of.

**Supplementary Information:**

The online version contains supplementary material available at 10.1186/s12879-024-09433-7.

## Background

Incubation time is the period from infection to symptom onset. Knowing its distribution is relevant to make decisions about public health measures as well as to parameterize mathematical models for disease spread. For the SARS-CoV-2 virus, the right tail of the distribution played a crucial role in determining the appropriate duration of quarantine. Estimation of the incubation time distribution of an infectious disease is hampered by incomplete data about infection. While time of symptom onset is usually known, the time origin is not. Typically, the only information available is a range of potential exposure times, yielding data with interval censored time origins. Insights into transmission are primarily obtained via contact tracing, where individuals with confirmed infection are asked about potential sources of transmission. As such, infectors and infectees can be traced.

While methods to estimate a distribution based on interval censored endpoints are well established, estimation with interval censored time origins is less straightforward. A commonly made assumption in SARS-CoV-2 incubation time estimation is that the infection time is uniformly distributed within the exposure window [29]. Then the likelihood with interval censored time origins can be written as the likelihood for interval censored endpoints. In an earlier study, we quantified the bias introduced when the uniform assumption is violated by means of a simulation study [1]. We found that the incubation time is overestimated if the infection risk increases rather than remains constant within an exposure window, as happens during the initial outbreak phase of a novel pathogen. To limit bias, analysis is often restricted to observations with narrow and well-defined exposure windows [18]. For instance, from the 255 first PCR confirmed cases of mpox in Italy, only 30 observations were used to estimate incubation time [11]. These observations were chosen because both a narrow period of exposure and symptom onset could be identified. This would not be a problem, had the observation been a random subset of the data. However, this selection of observations may introduce another bias, due to the presence of differential recall.

In order to inform policy makers with respect to prevention measures at the start of an outbreak, a rapid assessment of the incubation time distribution is needed. For SARS-CoV-2, these estimates were based on data from individuals who became infected in Wuhan, travelled from Wuhan right before the lockdown started, and developed symptoms after departure [2]. This means that their exposure window ended on the day of travel. Such data may be subject to two forms of length biased sampling. Right truncation occurs when individuals are omitted due to their ongoing incubation at the time of data collection, leading to under-representation of longer incubation times [17]. Left truncation occurs because data from Wuhan travellers only included individuals who developed symptoms after departure, leading to an under-representation of shorter incubation times. To the best of our knowledge, occurrence of left truncation in this context has not been described elsewhere.

This paper explores two biases that have been overlooked in the estimation of the incubation time for SARS-CoV-2: differential recall and left truncation. The structure of the paper is as follows. “Likelihood and commonly made assumptions” section introduces the likelihood and commonly made assumptions. “Literature” section discusses the literature on differential recall and left truncation in the presence of interval censored time origin. “Simulation setup” and “Results” sections present the simulation scenarios and results. The paper ends with a discussion where findings and their implications are presented. Practical recommendations for incubation time estimation are provided.

## Likelihood and commonly made assumptions

**Fig. 1:**
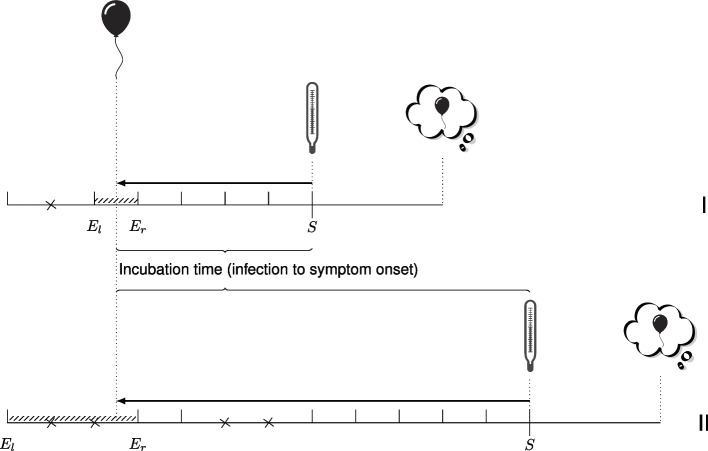
Illustration of memory decay. Graphical representation of incubation time and differential recall for two individuals I and II, both infected at the same party. During an interview conducted three days after symptom onset, both individuals were asked to recall their risk exposures. Individual I had a shorter incubation time (infection to symptom onset *S*) than individual II and therefore was exposed closer to symptom onset. In the simulation setup, decay of memories was mimicked by generating daily monitoring times (vertical tick lines) that may be forgotten (crosses) with certain probability as explained in the text. The observed exposure window consists of the last monitoring time before infection ($E_l$) and the first monitoring time after infection ($E_r$) that are not forgotten

Denote by *E* the time of infection. Typically, the knowledge about *E* is limited to an exposure period within which the infection took place, or only the end of the exposure period is known. We denote the start and end of the exposure window by $E_l$ (left) and $E_r$ (right) respectively, with $E_l$ possibly missing. The onset of symptoms (*S*) is usually known up to the precise day. We denote these events by upper case letters (*E* and *S*) and their realizations (*e* and *s*) by lower case; all given with respect to calendar time. An observation of incubation time consists of $(e_{il},e_{ir}, s_i)$ (see Fig. 1). Let $g_i(\cdot |e_{il},e_{ir})$ represent the individual-specific density of the infection time, having $[e_{il},e_{ir}]$ as support. Denote by $f(\cdot )$ and $F(\cdot )$ the density and the cumulative distribution function of the incubation time $T=S-E$, and let $h(\cdot ,\cdot )$ denote the density of the observation points that define the start and end of the exposure window.

Three assumptions are commonly made:

- The start and end of the exposure window are independent of the incubation time, i.e. $(E_{il},E_{ir}) \perp T_i$.
- The individual’s risk of infection is constant within the exposure window, i.e. $E_i|(e_{il},e_{ir}) \sim \textrm{Unif}(e_{il}, e_{ir})$.
- The distribution of the incubation time follows a parametric distribution, such as gamma, lognormal and Weibull.

Under assumption (a), the contribution to the likelihood for individual *i* is given by:

1 $$\begin{aligned} l(e_{il},e_{ir}, s_i) = h(e_{il},e_{ir})\int _{e_{il}}^{e_{ir}} g_i(u|e_{il},e_{ir})f(s_i - u)du. \end{aligned}$$

Note that although $E_{il}$, $E_{ir}$ and $S_i$ are commonly observed up to a specific day, this discretization is not accounted for in the likelihood.

It is challenging to verify the validity of assumption (a) since the moment of infection, and hence also the incubation time, are typically not precisely observed. We therefore rely on reasoning why (a) is valid. Observations of incubation time are usually collected retrospectively through interviews with diagnosed individuals. Suppose that at the beginning of an outbreak, individuals who developed symptoms are interviewed on the day of symptom onset (*S*). Then, a person with a long incubation time needs to recall an exposure that occurred longer ago compared to a person with a short incubation time. Assumption (a) is violated if some individual characteristics that on average increase incubation time and decrease recall ability are present. However, if the ability to recall possible exposure decays over time before symptom onset similarly for all individuals, assumption (a) still holds. We provide further details in “Differential recall” section.

Assumption (b) is convenient because it makes the likelihood proportional to a likelihood for interval censored end points

2 $$\begin{aligned} l(e_{il},e_{ir}, s_i) \propto F(s_i-e_{il})-F(s_i-e_{ir}). \end{aligned}$$

Standard estimation approaches and software are available with such interval censored end points. Assumption (b) is violated during the epidemic growth phase, leading to moderate bias in the estimates [1]. The amount of bias depends on the width of the exposure windows. Wider intervals do not necessarily result in greater bias. Specifically, individuals with very wide exposure windows that end at the time of symptom onset, do not provide any information. Individuals with a narrow exposure window contribute more to the estimate.

Assumption (c) is unrealistic. Historically, a lognormal distribution was commonly assumed, but the validity of the rationale behind this choice is nowadays considered questionable [21]. Whether this choice is problematic depends on the quantity of interest. The mean or median value will often be little affected by an incorrect choice of the parametric distribution of the incubation time. If the focus is on the estimation of a tail percentile, it becomes crucial to consider more flexibility in the choice of distribution. Coronaviruses are known to have an incubation time distribution with a long tail [35]. Hence, the gamma, lognormal or Weibull distribution may not adequately capture the true shape of the tail [35]. This issue can be partially overcome by using a semiparametric approach [1].

## Literature

### Differential recall

When collecting exposure information retrospectively through interviews with diagnosed individuals, it is important to keep in mind that our memory is not flawless. Recall bias is a term encompassing all sorts of biases that arise from differences in recall among participants in retrospective studies. A well-known example of recall bias is observed in case-control studies and retrospective cohort studies when estimating the risk associated with an exposure [20]. Cases tend to remember exposure status more accurately than controls. This misclassification inflates odds ratios and can lead to erroneous associations [14, 28]. However, differential recall is not limited to case-control studies but may occur in all observational data [20].

Several papers on estimating SARS-CoV-2 incubation time highlight recall bias as a problem [3, 36]. A systematic review and meta-analysis based on 42 studies where the aim was to determine the incubation period of COVID-19, showed that 78.6% (*N* = 33) of the estimates were potentially affected by recall bias [7]. Note that recall bias may occur in these studies as these typically rely on data obtained by *backward* tracing of potential infectors [4], e.g. tracing potential infectors. Recall bias is less likely to occur in data collected by *forward* tracing, e.g. tracing contacts that a notified case might have infected, but this practice is less common.

Memory of an event is worse if it happened longer ago (this phenomenon inspired the game “Match the Memory”). This has been observed for exposures in cases of foodborne Hepatitis A [25] and prion disease [31], that tend to have a long incubation time.

It may also be, that the timing of the event is remembered without systematic bias, but with less precision when it occurred further in the past. In the context of estimating the incubation time distribution, individuals with confirmed infection are asked by public health officials to report their potential past exposures at the time of the interview. Typically, individuals recall recent exposures more accurately than those that occurred further in the past, leading to a broader exposure window being reported. We call this differential recall.

The two definitions are provided here:

**Recall bias** *Umbrella term encompassing various biases that arise from differences in recall ability among participants in retrospective studies.*

**Differential recall** *The phenomenon that individuals exhibit less precise recollection of the timing of an event if the event occurred further in the past. In the context of incubation time estimation, this event typically refers to potential risk exposure.*

Differential recall does not necessarily introduce bias. It becomes problematic if researchers choose to restrict the analysis only to observations with “well-defined” exposure [18], where well-defined means that the exposure is either observed exactly or it falls within a narrow exposure window. Reasons for this choice are ample, such as: considering these observations to be more reliable; attempting to limit bias if a constant risk of infection over time is assumed [1]; or simplifying the analysis by treating exposures as exact rather than interval censored observations. When there is differential recall, restricting the analysis to observations with well-defined exposure may introduce bias, because observations with shorter incubation times tend to have shorter exposure windows and therefore are more likely to be included.

There is no differential recall if the exposure windows are based on test results. One example is estimation of the HIV incubation time distribution based on data from cohort studies in which individuals are tested for HIV infection at each visit [10].

Literature on memory decay and differential recall is scarce, and studies typically do not concern the infectious disease context. Most studied the strength of memory decay. Literature in experimental psychology suggests that memory decays exponentially with time [33]. Two studies found that the recall of injuries declined if they happened longer before the interview [13, 19]. Since these studies did not consider respiratory infection and considered recall aggregated by month, results cannot necessarily be extrapolated to the SARS-CoV-2 setting. In elderly, the recalled fall rate showed a decline of 9% in a one-year compared to a quarterly survey [37]. Two studies describe differential recall of age at menarche [32, 34]. Their data include a combination of observations with exact event times and current status data, where the age at menarche is left- or right-censored. The probability of recall, i.e. exactly observing the age at menarche, is assumed to depend on the time between menarche and the moment of recall, and it is modeled with a piecewise function.

One study focused on the mechanisms of differential recall, and discusses methods to improve the responses [33]. Note that their conceptualization differs from differential recall as we stated in our definition. In the analysis of the impact of memory decay on responses in surveys, the authors propose a model for the effect of time on memory in survey interviews. This model consists of two components: forgetting an exposure entirely or placing it more recently than it actually occurred, which is known as forward telescoping. The latter was observed to occur more frequently than misplacing the exposure in the opposite time direction (backward telescoping). In survey research, Weber’s law [12] describes the error in time perception due to telescoping as a function of the logarithm of the time period.

Other directions to mitigate bias due to differential recall relate to the interview process [33]. The following techniques may be beneficial for memory responses:

- Use of records: this involves providing records of event details.
- Aided control: by providing specific cues, such as using pictures or lists of possible exposure locations or using aided recall questions like “Did you visit a grocery store, and if so, when?”;
- Bounded recall: conducting a series of interviews covering bounded time periods (e.g. biweekly, focusing on the last two weeks).

Additionally, they discussed how interview characteristics can influence recall bias. These factors include whether it is self-administered or face-to-face, the positioning of questions, and the type of questions (open or closed).

McAloon et al. warn that the subset of observations with well-characterized exposures for SARS-CoV-2 may be biased toward more severe cases [18], thus violating assumption (a). If severe cases tend to have shorter incubation periods [15], the estimates may be biased downward.

Determining the presence of differential recall in the data is challenging due to the unknown exact moment of infection. Ideally, one would assess the correlation between the width of the exposure window and the incubation time to quantify the extent of differential recall. As an approximation, the interval between the end of exposure and the interview date can be used instead of the incubation time. If a strong positive correlation is identified between the exposure window width and this interval, it serves as an indication of the presence of differential recall in the collection of exposure information.

### Left truncation

The initial studies using data from Wuhan only included individuals who left Wuhan before the lockdown started (January 23, 2020) and were free of symptoms until the day they left Wuhan. Consequently, individuals with shorter incubation times were more likely to be excluded.

Apart from *E* and *S* as the calendar time of infection and onset of symptoms respectively, we additionally denote *V* as the calendar time of leaving Wuhan. The observed data for individual *i* are $(e_{il},e_{ir}, v_i, s_i)$ where individual *i* is included in the analysis because $v_i < s_i$. For many individuals $e_{ir}=v_i$. In the likelihood specification, this leads to a denominator term that quantifies the probability to be free of symptoms at the time of leaving Wuhan. Let $h'(e_l,e_r,v)$ denote the joint density of the observation points around the moment of infection and the time of leaving Wuhan.

Then the likelihood provided in (1) is replaced by

3 $$\begin{aligned} l(e_{il},e_{ir}, v_i, s_i| v_i < s_i) = \frac{h'(e_{il},e_{ir},v_i)\int _{e_{il}}^{e_{ir}} g_i(u|e_{il},e_{ir})f(s_i - u)du}{\int _{e_{il}}^{e_{ir}} g_i(u|e_{il},e_{ir})[1-F(v_i-u )]du}. \end{aligned}$$

Currently, there is no suitable R package available for this specific type of survival data. Pak et al. consider a similar type of data structure. They postulate a distribution for the time from infection to enrollment $V-E$ with density *k* [23]. Assuming that $V-E$ and *T* are independent, the following likelihood is obtained

$$\begin{aligned} l(e_{il},e_{ir}, v_i, s_i| v_i < s_i) = \frac{h(e_{il},e_{ir})\int _{v_i-e_{ir}}^{v_i-e_{il}} k(u)f(u+s_i-v_i)du}{\int _{v_i-e_{ir}}^{v_i-e_{il}} k(u)[1-F(u)]du}. \end{aligned}$$

They applied the data to a cohort study on HIV infection, allowing for right censored data with respect to symptom onset.

Qin et al. rightly acknowledge that the sampling mechanism of traveler data from Wuhan introduces length biased sampling [26]. They treated the incubation period as a renewal time and the duration from departure to symptom onset as forward time in a renewal process. This approach it is not suitable for our specific context [1].

## Simulation setup

We performed a simulation study to investigate the effects of differential recall and the presence of left truncated data. To examine differential recall, we varied: the strength of differential recall; whether the complete data or a subset was used in the analysis. To investigate how the presence of left truncated data affects the results, the following aspects were changed: the width of the exposure window; the distribution of infection risk: constant, increasing, or decreasing; whether to account for the presence of left truncation.

### Data generation

In the following sections, we provide details about how the data were generated to study the effect of differential recall and the presence of left truncated data, on the estimate of the incubation time distribution. In each scenario, the incubation time (*T*) was generated from a Weibull distribution with parameter values based on estimates for SARS-CoV-2 during the early stages of the pandemic (median 5.4 days, 95th percentile 9.8 days) [16]. Since we were not interested in the bias due to an incorrect parametric model, a Weibull distribution was assumed for estimation as well. One thousand data sets were generated in each scenario.

#### Differential recall

The basic idea of the data generation is sketched in Fig. 1. For each individual *i*, we first generated a sequence of daily monitoring times, spanning from 1 to 20 days before the onset of symptoms (indicated by vertical tick marks). This approach builds upon the work of Dejardin and Lessaffre [5]. These monitoring times act as observation points concerning infection status. They are forgotten with a certain probability (represented by crosses). As we remove observation times, we end up with a new, wider, exposure window. This means that we cannot employ the data generation approach used in our previous work, where we initially generated infection times uniformly within an exposure window and then generated an incubation time [1]. Instead, we generated times from symptom onset backwards to infection time as indicated by the arrow in Fig. 1. This process allowed us to directly create interval censored time-to-event data, and we do not need to assume a constant infection risk within the exposure window.

Regarding incomplete memory, we assumed that forgetfulness increases as individuals have to look further back in time. The probability of missing a monitoring time increases as the exposure time moved further away from symptom onset. More specifically, the probability varies with the timing of the monitoring moment, but remains the same across individuals. Additionally, we generated a subset of individuals (10%) with perfect recall of the time of infection, to guarantee that the models we fit later on are identifiable.

The probability to remember was modeled as $e^{-\lambda d}$, where the parameter $\lambda$ represents the differential recall rate and *d* the number of days that elapsed since the monitoring time at the interview day. Different values for the strength of $\lambda$ were used. We explored two estimation approaches: one that uses the complete data set (*N* = 500), while the other restricts the analysis to exposure windows narrower than 5 days, which we will refer to as the “subset” approach.

We describe how we obtained exposure information in the simulations using Fig. 1, which illustrates the timelines of two individuals. Both individuals are infected (indicated by balloon) at the same event but individual I develops symptoms (indicated by thermometer) soon after infection, while individual II has a much longer incubation period. Upon diagnosis, both are asked to recall their risk exposure. Individual I was exposed more recently at the time of interview (indicated by thinking cloud).

We generated daily monitoring times represented by vertical tick lines. Memory decay is incorporated by considering a probability of omission (indicated by crosses) that increases as the monitoring times are longer ago. The observed exposure window consists of the two memorised monitoring times closest to the moment of infection ($E_l$ and $E_r$). In the example in Fig. 1, the exposure window of individual II is wider than of individual I.

#### Left truncation

We generated data in a similar way as in our earlier paper [1], but with a different selection of exposure windows. Ten per cent had the moment of infection (*E*) observed exactly and they travelled on the day of infection, while the remaining 90% all had the same width of the exposure window (0 to $E_r$ where $E_r$ represents the preset width). This choice was made to guarantee that the models we fit later on are identifiable and to mimic the realistic scenario that some individuals were exposed during their travel day only (interpreted as exact observations for simplicity) [6]. We varied the width of the exposure window among scenarios. Next, we generated the time of infection (*E*) within the exposure window, an incubation time (*T*) and a time of symptom onset ($S=E+T$). This generation process made three different assumptions with respect to the time of infection within the exposure window:

- A constant risk of infection ($g(t) \sim U(E_l, E_r)$);
- Exponential growth with a five-day doubling time of the incidence ($g(t) \propto e^{0.14t}$), which reflects the initial phase of the outbreak in Wuhan [8];
- A declining infection risk ($g(t) \propto p(1-p)^{t-1}$ where $p = 0.2$ on the interval $[E_l, E_r]$), which may represent household transmission.

**Fig. 2:**
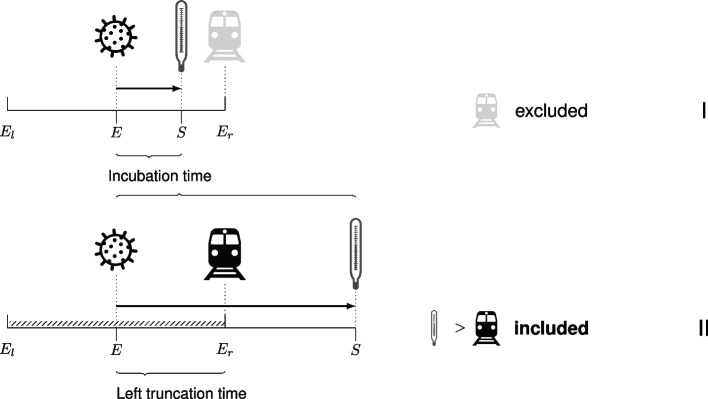
Illustration of left truncation. Two individuals were infected on the same day during the outbreak in Wuhan. Individual I had a shorter incubation time (infection to symptom onset, *E* to *S*) than individual II. Individual I and II planned to leave Wuhan at the same calendar date. However, individual I developed symptoms before the travel day; individual II developed symptoms after leaving Wuhan. Individual II is included in the data concerning travellers from Wuhan, with a left truncation time (interval) from infection (*E*) to travel day ($E_r$). Individual I is excluded from the data

We only included individuals who experienced symptom onset after the end of the exposure window, i.c. after leaving Wuhan ($S> E_r=V$). This leads to left truncated data as illustrated in Fig. 2. In the figure, individual I had a shorter incubation time than individual II. Individual I developed symptoms (indicated by thermometer) before their scheduled departure from Wuhan (indicated by train), and remained in Wuhan. In contrast, individual II traveled while incubating and developed symptoms later. Individuals with $T<E_r-E$ were discarded. In this example, it means that individual I is excluded from the data, while individual II is included, i.e. observed in the data.

For each data set, we initially generated 50,000 observations, and then a random sample of 500 observations was selected satisfying the condition $S> E_r$.

### Estimation

The method used for data with differential recall yields data sets containing exact, interval censored and right censored observations. Observations are right censored when there is no memorised monitoring before infection, which may occur because we limited the maximum number of monitoring points to 20. The R package survival was used to fit the appropriate models to these data sets, assuming a Weibull distribution.

For the simulations with left truncated data, we use the reversed time scale, which assumes a constant risk of infection. We also assume that the time of infection is known for the truncated part of the likelihood, which is not the case in practice. Hence instead of Eq. (3), we maximized the “oracle” likelihood based on

4 $$\begin{aligned} l'(e_{il},e_{ir}, s_i| e_{ir} < s_i) = \frac{F(s_i-e_{il})-F(s_i-e_{ir})}{1-F(e_{ir}-e_i )}. \end{aligned}$$

This is somewhat artificial and merely serves to illustrate the problem with left truncated data, rather than to provide an actual solution. As the survival package does not incorporate the combination of interval censoring and left truncation, the R package MixtureRegLTIC was used to fit a time-to-event model (accelerated failure time model, AFT) to the data. The latter uses the extended generalized gamma (EGG) distribution, which was introduced by Farewell and Prentice [9] and includes the Weibull distribution as a special case.

### Performance measures

The performance of the model across 1000 estimates of the median and 95% percentile of the incubation time distribution per scenario is summarized by the bias (i.e. the average) and the interquartile range (p25 and p75) of the deviations between true and estimated value. Additionally, for the simulations concerning left truncated data, the mean proportion of exact observations in the resulting data sets is provided.

While all runs for the data sets with differential recall provided a model fit, for the scenarios concerning left truncated data, the model did not converge for some of the runs. This issue is due to an artifact inherent in the simulation setup. Specifically, it may occur that an observation has a late entry time that exceeds the lower bound of the interval censored incubation time, i.e. $s_i-e_{ir} < e_{ir}-e_i$ in Eq. (4). The MixtureRegLTIC software package was designed for observations with exactly observed time origin, interval censored endpoints and left truncation with respect to the endpoint. The percentage of invalid runs is shown in Supplement 2 and indicated by ‘Inv.’.

### Software

All analyses were performed in R version 4.1.1 [27] and R Studio version 2021.09.20 (“GhostOrchid”) [30] software environment, using the computing resources from the Academic Leiden Interdisciplinary Cluster Environment (ALICE) provided by Leiden University. The analysis code can be accessed via www.github.com/vharntzen/TwoBiasesExposure.

## Results

All performance measures can be found in the tables in Supplement 1 and 2.

### Differential recall

**Fig. 3:**
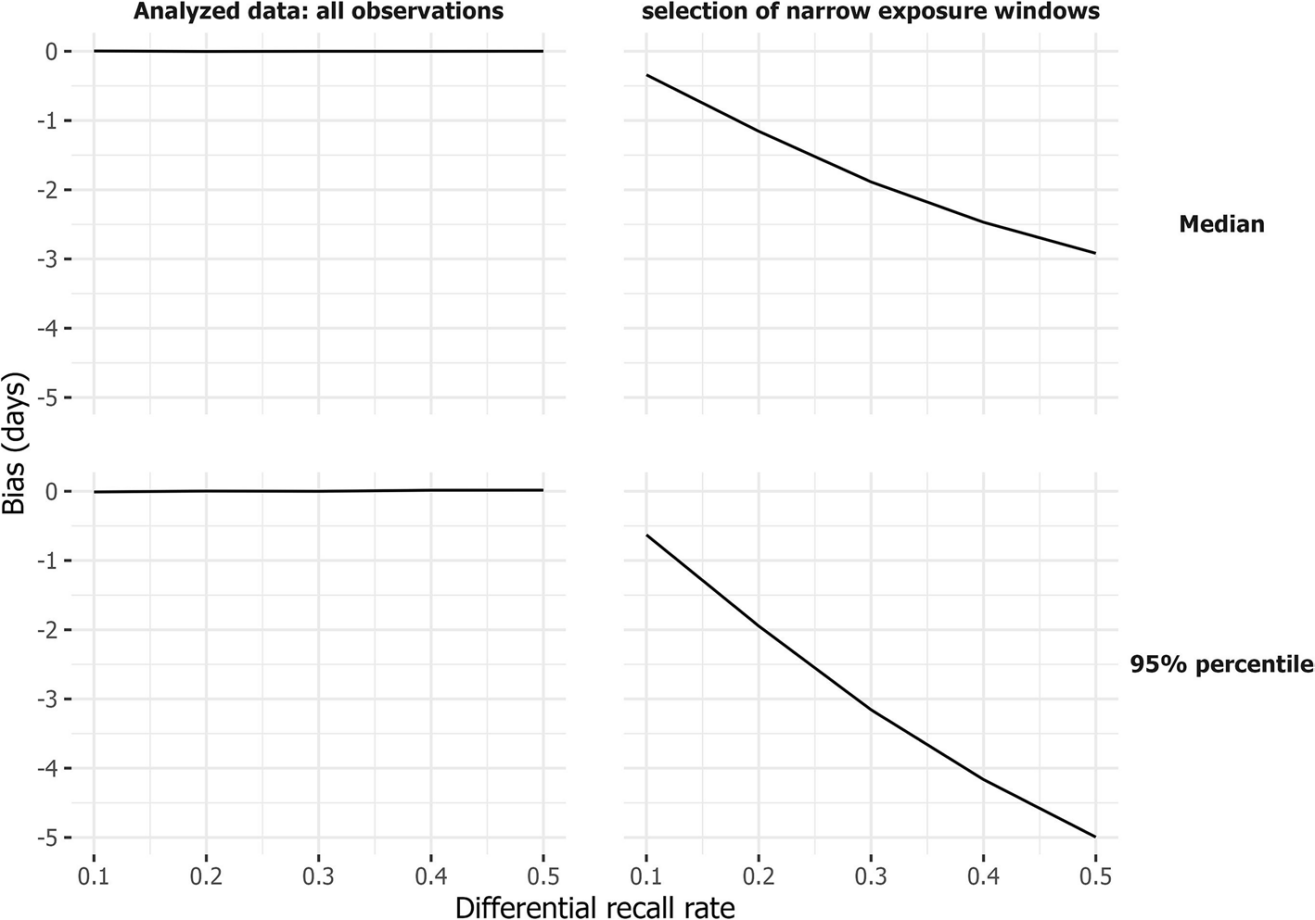
Results of simulations concerning differential recall. The bias (y-axis) is presented, based on 1000 generated data sets, for the estimated medians (upper panel) and 95^th^ percentiles (lower panel) under different strengths of differential recall (x-axis). Two analysis approaches are considered: one using all observations (left panel) and the other using a selection of narrow exposure windows (window width $\le$ 5 days, right panel). The recall probability per monitoring time, as depicted in Fig. 1, depends on backward time from symptom onset

Figure 3a visualizes the bias (y-axis) resulting from memory decay for different percentiles (upper row: median; lower row: 95^th^ percentile) and approaches (columns). These approaches include analyzing all observations (left panels) and analyzing a selection (right panels).

Estimates of the median and 95th percentile are unbiased, meaning that the average difference between the true and estimated number of days is close to zero, when all data is analyzed (Fig. 3a, left panel). This holds regardless of the rate of memory decay, since the distribution of non-omitted observation times is independent of the incubation time.

Using only observations with well-defined exposure (window width $\le$ 5 days, on average 39% of the observations for a differential recall rate of 0.3, for both scenarios) gives a similar downward bias (Fig. 3a, right panel). Individuals with longer incubation times tend to have wider exposure windows. Therefore, restricting to narrow windows selectively includes those with shorter incubation times. The magnitude of this bias increases with more extreme levels of differential recall.

### Left truncation

**Fig. 4:**
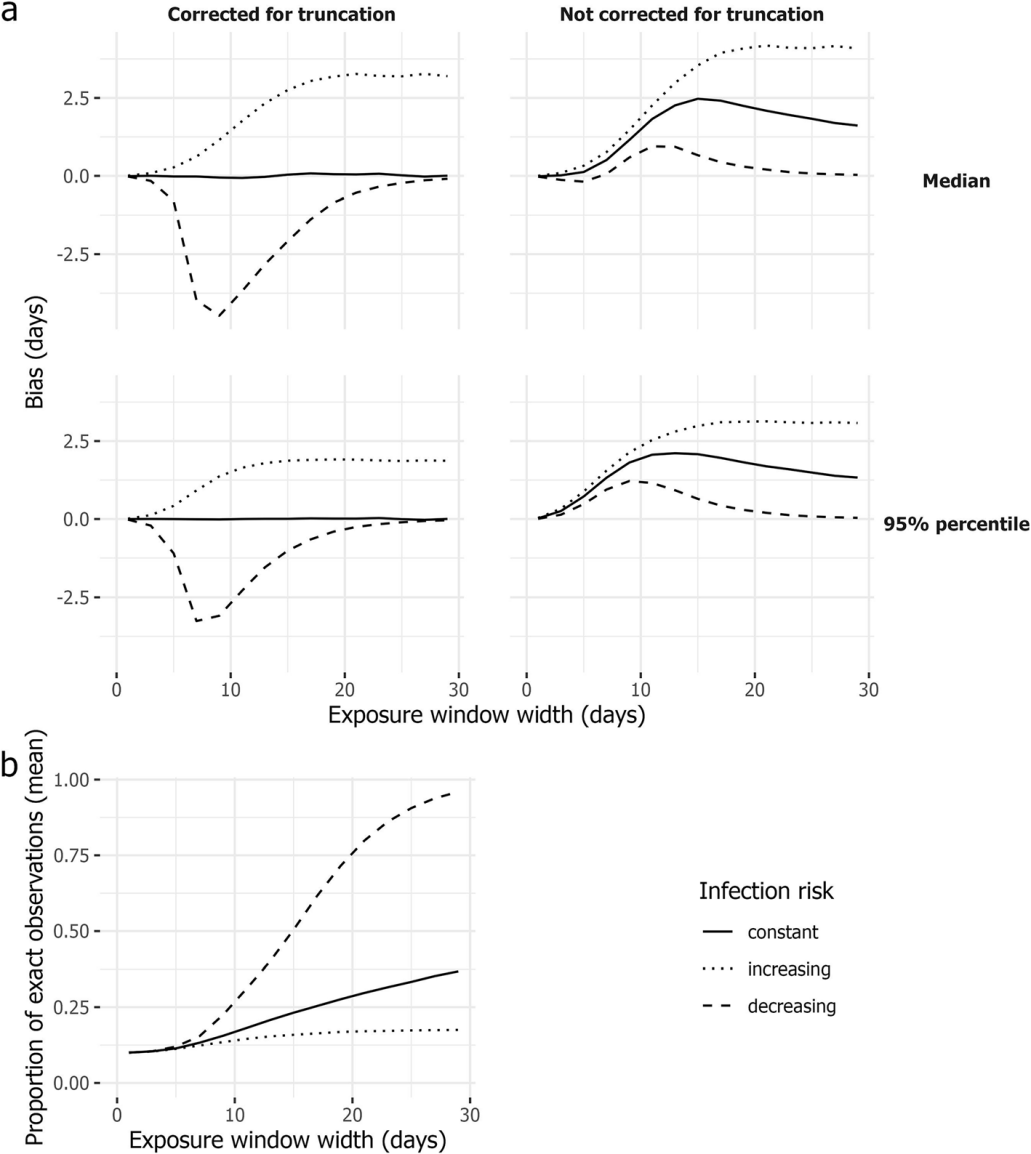
Simulation results concerning left truncation. **a** The bias (y-axis) is presented, based on 1000 generated data sets, for the estimated medians (upper panel) and 95^th^ percentiles (lower panel) across various exposure window widths (x-axis; not applicable to the initial 10% of observations with exactly observed moment of infection). Three different scenarios for the risk of infection within the exposure window are considered: constant (solid lines); increasing (dotted line) or decreasing (dashed line). The analysis is performed with truncation incorporated (left panel) or without (right panel). **b** The mean proportion of exact observations (y-axis) in the data set used for analysis, for different exposure window widths (x-axis). The infection risk distributions (constant, increasing, decreasing) on the exposure window domain are represented by different line types

The bias, i.e. the average difference between true and estimated, when we corrected for left truncation in the analysis is shown in the left panel of Fig. 4, while the bias resulting from leaving truncation uncorrected is shown in the right panel. The figure visualises the results for different exposure window widths (x-axis), percentiles (upper panel: median; lower panel: 95^th^ percentile), and true infection risk distributions (line type).

When the risk of infection is constant on the exposure window (solid line) and left truncation is accounted for in the analysis (left panel), estimates are unbiased regardless of the exposure window width (x-axis). However, when left truncation is neglected (right panel), estimates exhibit an upward bias. This bias initially increases with exposure window width, followed by a decline until it appears to stabilize.

Under a decreasing risk of infection within the exposure window (represented by the dashed line), the bias approaches zero as the exposure window width increases. This is because only the non-truncated, exact observations remain for the analysis (travel on day of infection). The rationale behind this is as follows: infection is most likely to occur at the beginning of an individual’s exposure window. The wider the window, the less likely it is for symptoms to develop after the end of the exposure window rather than within it. Hence, with the left truncation mechanism in place, it is less likely for such an observation to be included. Note that the absolute bias in the right panel of Fig. 4 is smaller than in the left panel and it operates in the opposite direction. This difference is because the two components of bias in the right panel partially cancel each other out. There is an upward bias when left truncation is not accounted for and a downward bias due to the violation of constant risk of infection (assumption (b) in “Likelihood and commonly made assumptions” section). In the left panel of Fig. 4, only the latter component of bias is present (resulting in downward bias).

At the beginning of an outbreak, the cumulative infection incidence and, consequently, the risk of infection grows exponentially. When the risk is increasing, an upward bias is observed (as indicated by the dotted line in Fig. 4), regardless of whether we corrected for truncation (left panel) or not (right panel). This bias increases with window width as the constant risk assumption is more strongly violated. In contrast to a decreasing risk (dashed line), both components of the bias point in the same upward direction. Without correction for truncation (right panel), the bias is larger than when left truncation is addressed in the analysis (left panel). Since infection is most likely to occur right before the end of the exposure window, a substantial portion of the data is used for analysis even when the exposure window width is large, preventing the bias from vanishing. Moreover, as exposure windows get wide, the bias plateaus rather than vanishes. Note that the bias remaining after correction for left truncation (left panel) is the same bias as observed in previous work [1].

## Discussion

Incubation time plays a critical role in informing policy makers during the early stages of an outbreak. However, accurate estimation is challenging due to limitations in the data, which is typically collected retrospectively through interviews with infected individuals regarding their exposure. In this study, we investigated the impact of two phenomena in SARS-CoV-2 contact tracing data that have been neglected in estimation: differential recall of exposure and left truncation. Our simulations revealed that, under the plausible scenario where the start- and endpoints of the exposure windows are independent of incubation time differential recall does not introduce bias in the estimates when analyzing the complete data set. However, when the analysis is restricted to individuals with well-defined exposure which is often the case when observations are considered recall-biased, incubation time tends to be underestimated. Neglecting left truncation in the analysis consistently leads to overestimation.

The value of our study lies in recognizing the sources of bias involved in incubation time estimation. The phenomenon of differential recall may also occur in other contexts where time-to-event data is observed, such as environmental or work-related exposure to toxic agents and the subsequent development of health conditions.

Although right truncation has been mentioned in previous papers on SARS-CoV-2 incubation time estimation [17], left truncation has mostly been overlooked. Qin and Deng did consider left truncation in their analysis of the Wuhan data [6, 26]. However, we found that the method they proposed was not suitable for this particular context [1]. Since the exact moment of infection is not observed for most individuals, the same holds for the time from infection to entry into the study (i.c. leaving Wuhan). It is possible to adjust the likelihood to account for this specific problem by integrating over all possible infection moments within the exposure window. We explored the method proposed by Pak et al.  [24] and the corresponding R software that they provided upon request, but did not include it because it assumes a distribution for the time between infection and travel.

The concept of differential recall of time-to-event data has received little attention in the literature. Our simulations show that neglecting this phenomenon does not introduce any bias when the distribution of observation points (i.e., start and end of exposure) is unrelated to the time-to-event distribution and we use the full data set. While we consider this independence assumption plausible in the context of retrospectively collected contact information, verifying it in reality is difficult since the moment of infection is interval censored at best. Future research is needed to develop an algorithm capable of distinguishing whether this holds in real data. Note that in fact, window width may depend on the number of reported risk contacts as well. For example, while increased uncertainty in recall of an exposure leads to a wider exposure window, failing to memorize a specific contact may yield a narrower window instead. This issue about recording a specific contact was beyond the scope of our study, however it would be an interesting and useful future research direction. Our simulations revealed that restricting the analysis to narrow exposure windows introduces bias. It is important to note that the analysis is usually restricted to observations with narrow exposure windows for a valid reason, specifically to mitigate bias resulting from the violation of the constant risk assumption, particularly during the exponential growth phase of an outbreak. Apart from preventing bias due to differential recall, also including the individuals with wider intervals increases the size of the typically small data set, thereby increasing statistical power and narrowing the width of the confidence intervals.

Verifying the presence of phenomena as differential recall or an over-representation of long incubation times in real-world scenarios can be a challenging task; let alone to know the extent to which such factors bias the estimates when these are unaddressed in the analysis. Our second simulation study was motivated an overlooked source of bias in the analyses of the data from Wuhan in the beginning of the SARS-CoV-2 pandemic; however, the set of relevant phenomena differs per data set. A useful next step would be to examine ways to recognize those in real data. Moreover, different biases may cancel each other up to some extent. Further research is needed into how the different biases compare in terms of direction and size. Depending on the purpose of the estimate, underestimation of incubation time may be more harmful than overestimation that provides a conservative estimate.

A concern is that individuals with narrow exposure windows may not be representative of the entire population, but over-represent a group with shared characteristics such as a certain age, health status or attending a certain event with high transmission rates [18, 35]. If the incubation time distribution depends on such a characteristic, the resulting estimate is not representative for the entire population. An example is age, which was found to be related to memory in survey questions [33]. In that scenario, even analyzing all data would yield biased estimates as we saw in sensitivity analyses (not shown). Software for analyzing data with an interval censored time origin rather than endpoint, where a more realistic distribution of the infection risk within the exposure window can be used using a population-wide estimate of the infection incidence, would circumvent the need to assume a constant risk of infection. This would eliminate the need to restrict the analysis to a well-defined subset.

Our study offers practical recommendations for researchers involved in estimation of incubation time. Firstly, caution is warranted when restricting the analysis to observations with narrow exposure windows. While this reduces bias resulting from the potential violation of the constant risk assumption, it may lead to underestimation of the incubation time distribution due to differential recall. If there is doubt whether differential recall plays a role, a sensitivity analysis comparing results with and without wide exposure windows is recommended. Secondly, researchers need to be aware that left truncation may be present in the data. We gave the specific example of data on SARS-CoV-2 infection based on individuals that left Wuhan. A scenario other than traveller data in which this may occur, is when infected individuals experience a high case fatality rate, and are ascertained by screening. Individuals with a short incubation time may tend to have deceased already, such that exposure information cannot be obtained anymore via retrospective interviews.

In a more general context, obtaining optimal estimates of the incubation time distribution requires comprehensive retrieval of exposure information. Typically, this information is obtained through retrospective interviews with detected cases, and these interviews should cover a sufficiently long period to capture all potential risk exposures. If the period is too short, the true infection may not fall within the given exposure window. Additionally, when the case definition assumes only a narrow range of potential incubation periods, implying a limited exposure period, longer incubation periods may go unnoticed. To prevent the latter problem from occurring, the incubation time could be excluded from the case definition, but this increases the risk of misdiagnoses. In other words, a less specific case definition complicates diagnosis. For example, in the case of influenza and corona viruses, for which the clinical presentation shows strong similarities, including the incubation period in the case definition is useful for distinguishing between these respiratory infections [22].

Our study discusses two overlooked sources of bias in incubation time estimation, acknowledging that resolving them in practice may not be straightforward. We provide practical recommendations for researchers engaged in estimating incubation time.

### Supplementary Information

Supplementary Material 1.

## Data Availability

The code for data generation and analysis can be accessed via www.github.com/vharntzen/TwoBiasesExposure.
