# Supplementary material for: Two biases in incubation time estimation related to exposure
Source: BMC Infect Dis. 2024 Jun 3;24:555. doi: 10.1186/s12879-024-09433-7 (PMC11149330; doi:10.1186/s12879-024-09433-7)
Supplement: Supplementary file 1 — Supplementary Material 1. [file 12879_2024_9433_MOESM1_ESM.pdf]

Supplementary Materials (1)

Table 1: Simulations results concerning differential recall. Each row summarizes 1000 runs, each with data set size N = 500. *Abbreviations:* Differential recall rate (Recall rate); Percentile estimand (Perc.); Data for analysis (Data): all observations or the selection of observations with an exposure window width smaller than 5 days; p25/p75: percentiles of the distribution of deviations between true and estimated percentile.

Recall rate	Perc.	Data	Bias	p25	p75
0.1	50	all	0.00	-0.08	0.08
0.2			0.00	-0.09	0.08
0.3			0.00	-0.10	0.10
0.4			0.00	-0.14	0.14
0.5			0.00	-0.18	0.16
0.1		selection	-0.34	-0.42	-0.26
0.2			-1.16	-1.25	-1.06
0.3			-1.89	-1.99	-1.78
0.4			-2.47	-2.58	-2.36
0.5			-2.92	-3.04	-2.79
0.1	95	all	-0.01	-0.16	0.15
0.2			0.00	-0.20	0.21
0.3			0.00	-0.28	0.26
0.4			0.01	-0.39	0.38
0.5			0.02	-0.52	0.49
0.1		selection	-0.63	-0.78	-0.48
0.2			-1.95	-2.12	-1.77
0.3			-3.16	-3.36	-2.95
0.4			-4.16	-4.42	-3.92
0.5			-5.00	-5.29	-4.71

Supplementary Materials (2)

Table 2: Simulations results concerning left truncation. Each row summarizes 1000 runs, each with data set size N = 500. *Abbreviations:* Exposure window width in days (Width); Percentile estimand (Perc.); Infection risk distribution on exposure window (Risk): constant (cons.), exponentially increasing (incr.) or decreasing (decr.); mean proportion of exact observations in the final data sets (PE); p25/p75 quantiles of the distribution of deviations between true and estimated percentile; percentage of invalid runs, i.e. for which the model did not converge (Inv.).

Width	Perc.	Risk	PE	Corrected for truncation				Not corrected for truncation			
				Bias	*p25*	*p75*	*Inv.*	*Bias*	*p25*	*p75*	*Inv.*
3	50	cons.	0.10	0.00	-0.10	0.10	0.0	0.26	0.18	0.33	0.3
7			0.13	-0.01	-0.22	0.24	0.0	1.33	1.24	1.41	0.0
11			0.18	0.00	-0.29	0.32	1.7	2.06	1.94	2.19	0.0
17			0.25	0.02	-0.22	0.28	23.7	1.96	1.81	2.11	0.0
25			0.33	-0.01	-0.20	0.20	18.1	1.49	1.36	1.61	0.0
3		incr.	0.10	0.13	0.03	0.22	0.0	0.33	0.25	0.42	0.0
7			0.12	0.92	0.76	1.07	0.0	1.55	1.46	1.64	0.0
11			0.15	1.64	1.43	1.86	1.1	2.53	2.40	2.66	0.0
17			0.16	1.90	1.68	2.12	10	3.10	2.94	3.27	0.0
25			0.17	1.86	1.59	2.11	8.6	3.08	2.85	3.29	0.0
3		decr.	0.10	-0.21	-0.31	-0.11	0.0	0.14	0.06	0.21	0.2
7			0.15	-3.26	-3.82	-2.74	12.1	0.95	0.86	1.04	0.1
11			0.30	-2.29	-2.63	-1.95	98.3	1.16	1.05	1.26	0.0
17			0.62	-0.65	-0.78	-0.53	44.5	0.43	0.35	0.52	0.0
25			0.91	-0.10	-0.19	-0.02	11.1	0.08	0.00	0.16	0.0
3	95	cons.	0.10	0.00	-0.12	0.12	0.0	0.02	-0.11	0.13	0.3
7			0.13	-0.02	-0.19	0.18	0.0	0.51	0.38	0.64	0.0
11			0.18	-0.07	-0.43	0.38	1.7	1.82	1.68	1.98	0.0
17			0.25	0.08	-0.38	0.58	23.7	2.41	2.11	2.71	0.0
25			0.33	0.02	-0.32	0.38	18.1	1.83	1.57	2.07	0.0
3		incr.	0.10	0.09	-0.03	0.21	0.0	0.09	-0.02	0.22	0.0
7			0.12	0.62	0.49	0.75	0.0	0.76	0.64	0.89	0.0
11			0.15	1.76	1.54	1.99	1.1	2.26	2.10	2.41	0.0
17			0.16	3.04	2.68	3.44	10.0	3.94	3.61	4.28	0.0
25			0.17	3.19	2.62	3.76	8.6	4.09	3.58	4.55	0.0
3		decr.	0.10	-0.16	-0.29	-0.04	0.0	-0.13	-0.25	-0.01	0.2
7			0.15	-3.98	-4.87	-3.02	12.1	0.06	-0.07	0.19	0.1
11			0.30	-3.69	-4.18	-3.33	98.3	0.95	0.81	1.09	0.0
17			0.62	-1.39	-1.62	-1.14	44.5	0.44	0.29	0.59	0.0
25			0.91	-0.23	-0.35	-0.08	11.1	0.07	-0.05	0.2	0.0
